# The Pharmacological Action of Kaempferol in Central Nervous System Diseases: A Review

**DOI:** 10.3389/fphar.2020.565700

**Published:** 2021-01-13

**Authors:** Jéssica Silva dos Santos, João Pedro Gonçalves Cirino, Patrícia de Oliveira Carvalho, Manoela Marques Ortega

**Affiliations:** ^1^ Laboratory of Cell and Molecular Tumor Biology and Bioactive Compounds, Post Graduate Program in Health Science, São Francisco University (USF), Bragança Paulista, Brazil; ^2^ Laboratory of Multidisciplinary Research, Post Graduate Program in Health Science, São Francisco University (USF), Bragança Paulista, Brazil

**Keywords:** flavonoids, kaempferol, Alzheimer, Parkinson, ischemia stroke, epilepsy, glioblastoma

## Abstract

Kaempferol (KPF) is a flavonoid antioxidant found in fruits and vegetables. Many studies have described the beneficial effects of dietary KPF in reducing the risk of chronic diseases, especially cancer. Nevertheless, little is known about the cellular and molecular mechanisms underlying KPF actions in the central nervous system (CNS). Also, the relationship between KPF structural properties and their glycosylation and the biological benefits of these compounds is unclear. The aim of this study was to review studies published in the PubMed database during the last 10 years (2010–2020), considering only experimental articles that addressed the isolated cell effect of KPF (C_15_H_10_O_6_) and its derivatives in neurological diseases such as Alzheimer's disease, Parkinson, ischemia stroke, epilepsy, major depressive disorder, anxiety disorders, neuropathic pain, and glioblastoma. 27 publications were included in the present review, which presented recent advances in the effects of KPF on the nervous system. KPF has presented a multipotential neuroprotective action through the modulation of several proinflammatory signaling pathways such as the nuclear factor kappa B (NF-kB), p38 mitogen-activated protein kinases (p38MAPK), serine/threonine kinase (AKT), and β-catenin cascade. In addition, there are different biological benefits and pharmacokinetic behaviors between KPF aglycone and its glycosides. The antioxidant nature of KPF was observed in all neurological diseases through MMP2, MMP3, and MMP9 metalloproteinase inhibition; reactive oxygen species generation inhibition; endogenous antioxidants modulation as superoxide dismutase and glutathione; formation and aggregation of beta-amyloid (β-A) protein inhibition; and brain protective action through the modulation of brain-derived neurotrophic factor (BDNF), important for neural plasticity. In conclusion, we suggest that KPF and some glycosylated derivatives (KPF-3-O-rhamnoside, KPF-3-O-glucoside, KPF-7-O-rutinoside, and KPF-4′-methyl ether) have a multipotential neuroprotective action in CNS diseases, and further studies may make the KPF effect mechanisms in those pathologies clearer. Future *in vivo* studies are needed to clarify the mechanism of KPF action in CNS diseases as well as the impact of glycosylation on KPF bioactivity.

## Introduction

In 1930, a new chemical substance isolated from oranges was discovered by Prof. Albert Szent Gyorgyti from University of Szeged, Hungary; it was believed to be a new member of the vitamin family and initially identified as vitamin P but later recognized as a flavonoid (Kwon et al., 2004; Kim and Yun-Choi, 2008). The flavonoids are bioactive compounds belonging to the polyphenols, a group that can be found in all plants and known as secondary metabolites responsible for protecting plants against oxidizing agents such as ultraviolet rays, chemical compounds, and pollution (Calderón-Montaño et al., 2011). More than 6,000 varieties of flavonoids have been identified and knowledge on their antioxidant action is well consolidated (Jäger and Saaby, 2011). Moreover, since the 2000s, an increasing number of studies have highlighted many beneficial effects of flavonoids associated to their cardioprotective, anticoagulant antiplatelet, antibacterial, antiviral, antifungal, anti-inflammatory, antitumor, and antineuroinflammatory activity (Kwon et al., 2004; Kim and Yun-Choi, 2008; Jeong et al., 2009; Bai et al., 2011; Pick et al., 2011; Woo et al., 2012; Tronina et al., 2013; Lee et al., 2013; Wang et al., 2013; Choi et al., 2014; Kim et al., 2014; Park et al., 2014; dos Santos et al., 2014; Materska et al., 2015).

According to their chemical structure, flavonoids can be categorized into six classes, namely: anthocyanidins, flavan-3-ols, flavanones, flavones, flavonols, and isoflavones (Yoon and Baek, 2005). Flavonols are the most frequent flavonoid chemical structures and are present in almost two-thirds of Western societies’ diet, responsible for giving food color and flavor, preventing oxidation of fat, and protecting vitamins and enzymes (Yao et al., 2004). Kaempferol, quercetin, myricetin, and isorhamnetin are examples of that class of flavonoids (Rathee et al., 2009).

The vast majority of flavonoids can be found in nature in different chemical forms that can vary in their hydrogenations, hydroxylations, methylations, malonylations, sulfations, and glycosylations. In addition, in nature, when glycids are present in the flavonoids, they are called glycoflavonoids or glycosylated flavonoids. Glycidic substitutions include d-glucose, l-rhamnose, glucorhamnoside, galactose, lignin, and arabinose (Birt et al., 2001). In the absence of glycids, the structure receives the name of aglycone (Nielsen et al., 2006).

There are a variety of studies that address the promising effect of flavonoids on central nervous system (CNS) diseases (Table 1). However, little is known about the cellular and molecular mechanisms underlying the kaempferol (KPF) flavonoid effect in the CNS; also, the relationship between KPF structural properties and their glycosylation and the biological benefits of these compounds is not clear. Thus, the aim of the present study was to review the biological effects of KPF and its glycosylated derivatives, enabling a new view regarding the potential action of these molecules in different CNS diseases and clarifying aspects for future studies.

**TABLE 1:** Summary of *in vivo* and *in vitro* studies considering the action of kaempferol (KPF) and its derivatives in Alzheimer, Parkinson, ischemia, epilepsy, major depressive disorder, anxiety disorders, neuropathic pain, and glioblastoma.

Disorders	Molecule	Experimental model		Mechanisms	References
		*In vivo*	*In vitro*		
Alzheimer	KPF	*Drosophila* positive expression of β-A - 42		Decreased acetylcholinesterase activity	Beg et al. (2018)
	KPF		Microglia cell line - BV2	-Decreased NO, PGE 2, TNF-α, IL-1β and ROS production induced by LPS -Suppressed the expression of iNOS, COX-2, MMP-3 -Locked the TLR4 activation -Inhibited LPS-induced NF-κB activation and phosphorylation of p38 MAPK, JNK and AKT	Park et al. (2011)
	KPF	Ovariectomized female Wistar rats		-Increased levels of antioxidants GSH and SOD. -Decreased MDA, TNFα and cytochrome c	Kouhestani et al. (2018)
	KPF -3-O-rhamnoside		*Escherichia coli*	-Inhibited formation, extension, and destabilization of A-β fibrils in *Escherichia coli*	Sharoar et al. (2012)
	KPF		Mouse-derived hippocampal neuronal cells (HT22)	-Inhibited A-β induced toxicity in mouse-derived hippocampal neuronal cells (HT22)	Yang et al. (2014)
	Kaempferide (KPF 4′methyl ether)	Male Kunming mice		-Increased BDNF expression, which in turn allowed autophosphorylation of TrkB -Increased CREB phosphorylation in the hippocampus of DA model mice, generating a protective effect	Yan et al. (2019)
	KPF	Male Wistar rats		Decreased acetylcholinesterase activity	Zarei et al. (2019)
	KPF		NMR experiment	-Inhibited the 42-mer amyloid b-protein (Ab42) aggregation	Hanaki et al. (2016)
Parkinson	KPF	Male BALB mice		-Improved the lesion of nigrostriatal dopaminergic neurons -Inhibited the production of IL-1β, IL-6, TNFα -Decreased the levels of MCP-1, ICAM-1, and COX-2 -Suppressed the HMGB1/TLR4 inflammatory pathway -Protected of the blood-brain barrier integrity	Yang et al. (2019)
	KPF	nlrp3 KO mice		- Protected against LPS and SNCA-induced neurodegeneration -Inhibited NLRP3 inflammasome activation -Reduced of cleaved CASP1 and interruption of the assembly of the NLRP3-PYCARD-CASP1 complex. Autophagy in microglia	Han et al., 2019
	KPF	Adult male C57BL/6 mice		-Increased levels of striatal dopamine and its metabolites -Increased SOD and GSH-PX activity and reduced MDA	Li and Pu (2011)
	KPF		SH-SY5Y bone marrow cell line	-Neutralized ROS production -Apoptotic signal block mediated by the protein kinase JNK and p38MAPK -Induced the formation of mature and late autophagosomes in cells exposed to KPF	Filomeni et al. (2012)
Ischemia stroke	KPF	Sprague-Dawley rats		-Inhibited microglia activation -Inhibited the proinflammatory interleukins TNFα, IL-6, IL-1β, IL-5 and the proteins MCP 1 and ICAM 1 and inhibited phosphorylation of NF-kB p65 translocation -Protected the integrity of the blood-brain barrier	Li et al. (2019)
	KPF	Male mice C57BL/6		-Inhibited mitochondrial fission by inactivating DRP1 -Improved mitochondrial dysfunction of neurons -Enhanced autophagy	Wu et al. (2017)
	KPF-3-O-rutinoside KPF-3-O-glucoside	Male Sprague-Dawley		Similar effect for both molecules - Inhibited of microglia and astrocyte activation -Inhibited of STAT3 and NF-kB p65 phosphorylation and translocation of NF-kB p65 -Promoted BBB protection	Yu et al. (2013)
	Kaempferide-7-O-(4″-O-acetylrhamnosyl)-3-O-rutinoside	Male Wistar rats		-Decreased levels of MDA and high activity of GSH-PX and SOD -Inhibited phosphorylation of NF-kB p65, protein levels, ICAM-1, COX-2, iNOS, BAX2 and inhibited the cleavage of caspase 3 and caspase 9	Wang et al. (2016)
Epilepsy	KPF		MDCK-MDR1	-Inhibited the action of the gp-P transmembrane glycoprotein	Ferreira et al. (2018)
	Kaempferitrin and *Justicia spicigera Schltdl*	Swiss Webster mice and Wistar rats		-Delayed the onset of myoclonic crisis -Delayed onset of generalized seizure -Decreased mortality	González-Trujano et al. (2017)
	KPF derivative 3-O-[(E)-(2-oxo-4- (*p*-tolyl) but-3-en-1-yl]		Cell culture BV-2 mouse microglia and mouse-derived hippocampal neuronal cells (HT22)	-Inhibited the production of proinflammatory mediators such as TNFα, IL-6, PGE2 and nitrite -Reduced levels of proinflammatory proteins COX-2 and iNOS -Prevented phosphorylation of the NF-kB p65 subunit -Promoted the activity of the AMPK and Nrf2/HO-1 pathways	Velagapudi et al. (2019)
Major depressive disorder	KPF	Male mice C57 and CD1		-Increasing AKT/β-catenin cascade in the prefrontal cortex -Reduce concentration of inflammatory mediators IL-1β and TNFα	Gao et al. (2019)
	KPF-3-O-glucoside	Female Wistar rats		-Important antidepressant modulation -Reversed the insensitivity to the antidepressant imipramine	Garza et al. (2019)
Anxiety disorders	KPF	Albino mice Swiss		-Anxiolytic activity similar to diazepam	Gupta et al. (2018)
	KPF	Wistar rats	FAAH inhibitor screening assay kit	-Inhibit the enzyme FAAH -Increase in eCB activity -Presented reduced fear reaction as freezing response	Ahmad et al. (2020)
Neuropathic pain	*Ferula hermonis L.* and *Sambucus nigra L.*	Swiss-Webster-mice		-Exerted hypoglycemic activity in animals -Improved the peripheral nervous function of the animals	Raafat e El-Lakany (2015)
	*Alstonia scholaris*	Wistar rats		-To improve neuropathic pain induced in mice	Singh et al. (2017)
Glioblastoma	KPF		GL-15 glioblastoma cells	-Decreased expression of metalloproteinases MMP2 and MMP9 -Increased expression of laminin and fibronectin proteins -Promoted morphological changes suggestive of apoptosis	Santos et al. (2015)
	KPF		GBM8401	-Inhibited of these ERK, PKCα and NF-kB pathways was observed, which consequently inhibited the activation of MMP9 -Reduced of invasion and migration of tumor cells	Lin et al. (2010)

## Methods

A narrative review of the studies published in the PubMed databases in the last 10 years (2010–2020) was conducted. Experimental studies that addressed the isolated action of KPF (C_15_H_10_O_6_) and its derivatives in neurological diseases such as Alzheimer's disease, Parkinson, ischemia stroke, epilepsy, major depressive disorder, anxiety disorders, and glioblastoma were included. Studies that evaluated the action of plant extracts and synergistic effects among flavonoids were excluded from this research.

## Discussion

### Kaempferol: Structure and the Impact of Glycosylation on Their Bioactivity

KPF, known chemically as 3,5,7-trihydroxy-2-(4-hydroxyphenyl)-4H-1-benzopyran-4-one (PubChem, 2020), can be isolated from black, green, and mate herb teas (Matsubara and Rodriguez-Amaya, 2006), as well as from numerous common vegetables and fruits, including beans, cabbage, grapes, broccoli, strawberries, kale, gooseberries, citrus fruits, Brussels sprouts, grapefruit, apples, dry raspberry, and tomatoes, and from plants or botanical products commonly used in traditional medicine such as mums (*chrysanthemum spp.*), ginkgo biloba (*Ginkgo biloba L*), lime trees (*Tilia spp.*), Chinese milkvetch (*Astragalus mongholicus*), field horsetail (*Equisetum spp.*), moringa (*Moringa oleifera*), and the Japanese pagoda tree (*Sophora japonica*) (Rice-Evans, 2001; Calderón-Montaño et al., 2011).

**FIGURE 1:**
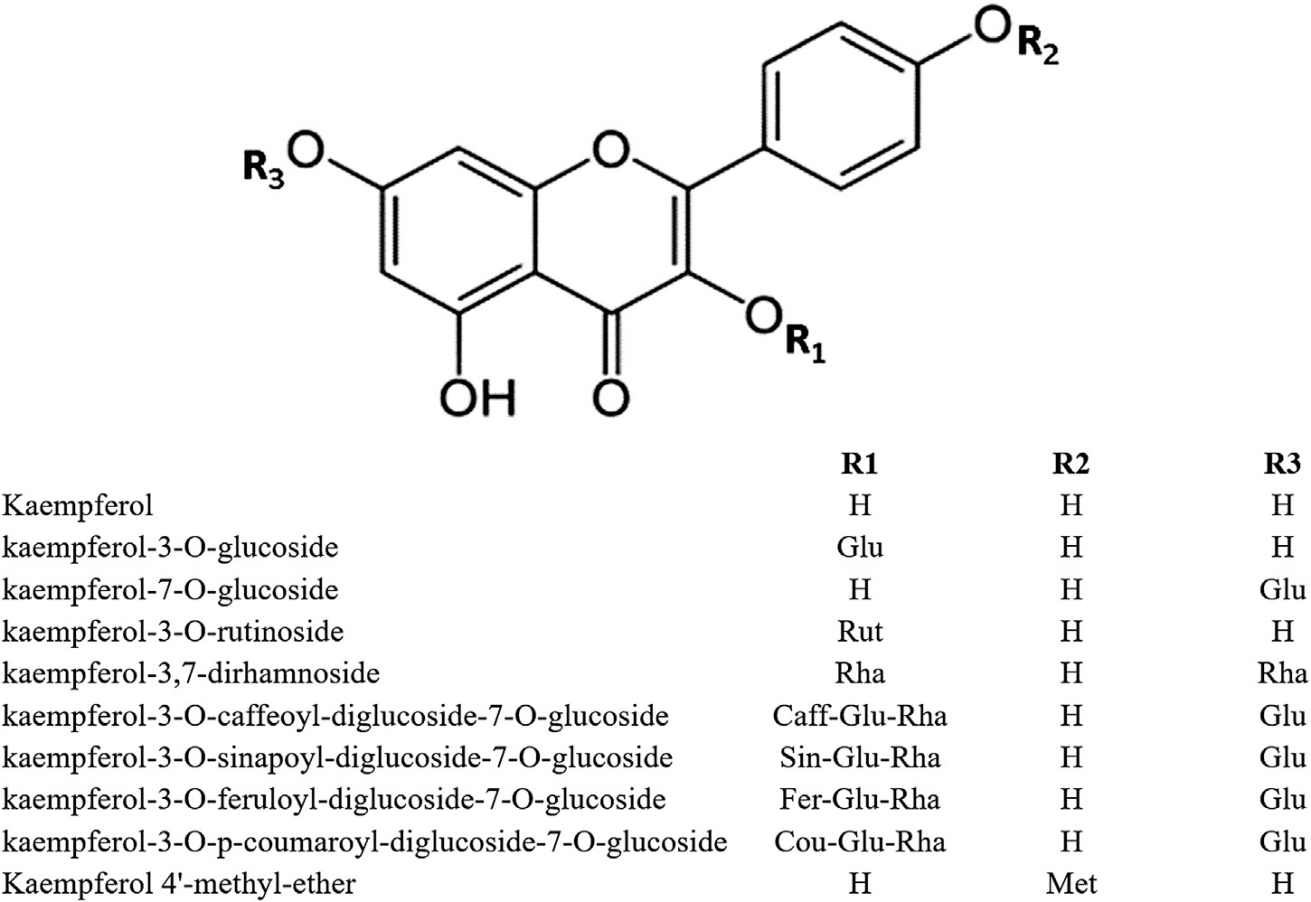
Structure of kaempferol and its derivatives.

In the plant kingdom, flavonoids are commonly found as glycosides. The most important KPF glycosides are astragalin (KPF-3-O-glucoside) and kaempferitrin (KPF-3,7-dirhamnoside). The *O*-glycosides of KPF can be acylated with hydroxycinnamic acids such as ferulic, sinapic, *p*-coumaric, and caffeic acids, as observed in KPF-3-O-caffeoyl diglucoside-7-O-glucoside, KPF-3-O-sinapoyl diglucoside-7-O-glucoside, KPF-3-O-feruloyl diglucoside-7-O-glucoside, and KPF-3-O-*p*-coumaroyl diglucoside-7-O-glucoside (Li et al., 2018) (Figure 1).

The absorption and bioactivity associated with different variations of the flavonoid molecule have been questioned and it has been observed that the bioactivity of glycosylated flavonoids *in vitro* can differ from that observed *in vivo* (Xiao, 2017). Although significant research has focused on the activity of KPF aglycone or its glycosides, few studies have compared their activities. Xiao (2017) has reviewed the influence of the glycosylation of flavonoids on their biological benefits, as well as the different pharmacokinetic behaviors among flavonoid aglycones and their glycosides. KPF appears to have higher antioxidant potential than its glycosylated derivatives. Shafek et al. (2012) have reported that the 2,2-diphenyl-1-picrylhydrazyl (DPPH) method free radical scavenging potential of two new KPF 3-O-glycosides (KPF 3-O-β-d–glucopyranosyl (1→2) β–d-xylopyranoside and KPF 3-O-α-l-arabinopyranosyl (1→2) β-d-galactopyranoside) from *Solenostemma argel* is weaker than that of KPF aglycone. Similarly, Wang et al. (2018), using DPPH and 2,2′-azino-bis (3-ethylbenzothiazoline-6-sulfonic acid) (ABTS) assays, showed that KPF had high free radical scavenging activity while its glycoside derivatives showed no significant activity.

Choi et al. (2002) have shown that glycosylation of KPF reduces its peroxynitrite scavenging potential and that KPF-3-O-glucoside has presented similar inhibitory activity to KPF-7-O-glucoside.

KPF and some of its glycosides can also significantly inhibit the production of nitric oxide (NO) and tumor necrosis factor-alpha (TNF-α) in RAW 264.7 cells stimulated by lipopolysaccharides (LPS) (Tran et al., 2009). Both *in vitro* and *in vivo* anti-inflammatory activities have been reported for KPF and KPF glycosides (Park et al., 2009). KPF can inhibit LPS and ATP-induced phosphorylation of phosphoinositide 3-kinase (PI3K) and serine/threonine kinase (AKT) in cardiac fibroblasts, thereby protecting cells from inflammatory injury (Tang et al., 2015).

KPF has presented stronger antiproliferative effect on HepG2, CT26, and B16F1 cell lines. In contrast, KPF-7-O-glucoside, KPF-3-O-rhamnoside, and KPF-3-O-rutinoside showed poor activity. Also, KPF efficiently inhibited T-cell proliferation and NO release when compared with its glycosides’ derivatives (Wang et al., 2018).

Among the various pharmacological effects presented by KPF, the anti-inflammatory action is the most striking, attributed mainly to its ability to inhibit the enzymes phospholipase A2, lipoxygenase, cyclooxygenase, and nitric oxide through the modulation of the enzyme nitric oxide synthase (iNOS) (Kim et al., 2004; Yoon and Baek, 2005; Santangelo et al., 2007). In addition, a previous *in vivo* study has proved the ability of KPF to overcome the blood-brain barrier (BBB) with a single dose of 600 mg/kilograms (kg) in rats (Rangel-Ordóñez et al., 2010).

### Pharmacological Action of Kaempferol in Central Nervous System Diseases

#### Alzheimer

Alzheimer's disease (AD) is a neurodegenerative disease clinically characterized by progressive loss of memory and cognitive functions (Hampel et al., 2018). In 2012, it was estimated that 24 million people worldwide were affected by AD and, given the rapid aging of the population in developed and developing countries, the frequency of cases will double every 20 years until 2040. In addition, AD is the most common cause of dementia in Western countries and still has no cure (Mayeux and Stern, 2012). It is not yet known why AD occurs; however, two characteristics are very common in the disease, namely, the presence of plaques generated by the accumulation of the abnormally produced beta-amyloid (β-A) protein and the hyperphosphorylation of the microtubule associated to the protein TAU, generating a neurofibrillary tangle, causing considerable loss of synapses, neuronal projections (dendrites), and, eventually, neurons (Serrano-Pozo et al., 2011). In addition, progressive loss of cortical interneurons and specific neurotransmitter pathways such as acetylcholine (ACh) is common in AD. ACh is an important neurotransmitter for memory consolidation and people with AD have low levels of ACh (Kumar et al., 2015; Ribeiro et al., 2017). One of the pharmacological approaches commonly used for the treatment of AD is the inhibition of the enzyme acetylcholinesterase, whose function is the hydrolysis of ACh in cholinergic synapses (Morsy and Trippier, 2018). The consequence of inhibition of this enzyme in patients with AD is that ACh is not destroyed so quickly and remains active for a longer time in the synaptic cleft, thus improving cholinergic transmission. However, AD still has no cure and treatment only slows the progression of the disease and relieves the symptoms of patients for an average of 9–12 months.

Studies have already shown that KPF has a neuroprotective effect; however, little is known about its neuroprotection mechanism. Beg et al. (2018) have demonstrated *in vivo,* using transgenic *drosophila* flies that express the β-A-42 protein, KPF's capacity to improve cognitive function through the inhibition of acetylcholinesterase activity, using an enriched KPF diet in different concentrations such as 10, 20, 30, and 40 µM for 30 days. Zarei and collaborators (2019) have also demonstrated the same effect in their study using Wistar rats AD models at dosages of 10, 20, and 40 μg per rat orally. In addition, both studies have shown the antioxidant action of KPF, which was responsible for decreasing neurotoxic motor and cognitive damage in *drosophila* flies and mice (Beg et al. (2018); Zarei et al., 2019).

Another study, using ovariectomized (OVX) rat models with streptozotocin- (STZ-) induced cognitive impairment, has shown that KPF was able to positively modulate the endogenous antioxidants, glutathione and superoxide dismutase (SOD), important against toxicity caused by reactive oxygen species (ROS) which contribute to the development of AD. Intraperitoneal injection of KPF (10 mg/kg) for 21 days was performed in the OVX + STZ + KPF group. Furthermore, KPF was able to decrease 21% of the formation of malondialdehyde, a lipid peroxidation marker, 52% of the TNFα, and 6% of the cytochrome C apoptosis marker in the hippocampus of the rats, explaining the decrease in neurotoxicity induced by STZ (Kouhestani et al., 2018).

Hanaki et al. (2016) have shown that 50 µM of KPF was able to inhibit β-A structure formation by preventing amyloid fibril elongation. Interestingly, the KPF derivative, KPF-3-O-ramnoside (C_21_H_20_O_10_), also showed similar effects *in vitro* using the cell line SH-SY5Y, resulting in the cytotoxic effect annulment generated by β-A (Sharoar et al., 2012). Similarly, Yan et al. (2019) have shown *in vivo* that the kaempferide (KPF-4′-methyl ether) (C_16_H_12_O_6_), another glycosylated KPF derivative, improves the expression of the brain-derived neurotrophic factor (BDNF) responsible for the formation of neural networks and neuronal plasticity. Those authors also observed that the improved BDNF expression induced autophosphorylation of tropomyosin kinase B, a BNDF receptor, and increased the phosphorylation of transcription factor cAMP, a response element-binding protein (CREB) involved in synaptic plasticity after presentation and memory formation.

Microglial activation is also a mechanism associated with several neurodegenerative diseases, including AD (Park et al., 2011; Zhang et al., 2013). One of the neuroprotection mechanisms exerted by different concentration of KPF (10–100 µM) was to suppress BV-2 microglia cells activation and its consequent neuroinflammatory toxicity by inhibiting the activation of inflammatory pathways such as toll-like receptor 4 (TLR4), factor nuclear kappa B (NF-κB), p38 mitogen-activated protein kinases (p38MAPK), and AKT (Park et al., 2011; Yang et al., 2014).

Currently, there is no definitive treatment for AD, since the AD signaling pathways are complex and the causal factors of the disease are unknown. Existing therapies improve some behavioral symptoms but hardly decrease cognitive deficit. In addition, at least half of AD patients have pharmacological resistance to the drugs currently available for treatment (Farlow et al., 2008). In fact, KPF has a multipotential neuroprotective action, given that its effect occurs through the modulation of several pathways involved with the progression of AD such as NF-κB, p38MAPK, and AKT. In addition, KPF has been shown to be effective in decreasing neuroinflammatory cytotoxicity, which makes this compound very attractive for in-depth studies that can better clarify its action.

#### Parkinson

Parkinson's disease (PD) is the most common age-related, chronic, neurodegenerative disease, affecting mostly people over 65 years old, with a worldwide prevalence around 1–2% (Dowding et al., 2006). The disease characteristic is the degeneration of nigrostriatal dopaminergic neurons and the presence of Lewy bodies (misfolded α-synuclein) in surviving neurons (Aarsland et al., 2017). The decrease in levels of protein tyrosine hydroxylase in the nigrostriatal dopamine region is also a characteristic frequently found in PD neurons (Aarsland et al., 2017).

A recent *in vivo* study using BALB mice has demonstrated that KPF was able to improve the lesion of nigrostriatal dopaminergic neurons; inhibit interleukin (IL) 1β, IL-6, and TNFα production; and decrease the levels of monocyte chemotactic protein-1 (MCP-1), intercellular cell adhesion molecule-1 (ICAM-1), and cyclooxygenase-2 (COX-2) (Yang et al., 2019). In addition, it was observed that KPF suppressed the HMGB1/TLR4 inflammatory pathway and ensured the protection of BBB integrity (Yang et al., 2019).

Han and collaborators (2019) have shown that KPF, at doses at 25, 50, and 100 mg per mice kg, was able to inactivate the NLRP3 inflammasome, which is constituted of NLRP3-PYCARD-CASP1 protein complex responsible for activating caspase-1 and mature IL-1β, thereby leading to an inflammatory response. Excessive activation of this complex generates several aging-related inflammatory disorders, including PD (Zhang et al., 2013). It was observed in mice studies that the inhibition of this complex by KPF resulted in inhibition of neurodegeneration, decreased secretion of IL-1β by microglia, and promoted microglial autophagy (Han et al., 2019).

Other studies have used mouse models for PD induced by neurotoxin 1-methyl-4-phenyl-1,2,3,6-tetrahydropyridine (MPTP) and KPF, at doses of 25, 50, and 100 mg per kg, as therapy, and the authors have observed that KPF improved motor coordination, increased levels of striatal dopamine and its metabolites, increased endogenous antioxidants SOD and glutathione peroxidase (GSH-PX) levels, and reduced malondialdehyde (MDA) levels (Li and Pu, 2011). More importantly, KPF was able to reduce common oxidative damage markers. The observed results coincide with the findings noted in AD patients (Li and Pu, 2011).

Filomeni et al. (2012) have demonstrated, in the bone marrow cell line SH-SY5Y exposed to rotenone, an odorless, colorless, crystalline isoflavone used as a broad-spectrum insecticide, piscicide, and pesticide, that KPF exposure, at concentrations ranging from 6 µM to 50 nM, neutralized rotenone-induced toxicity. Moreover, it reduced by half the number of apoptotic cells, mainly after 12 h of KPF treatment, by inhibiting caspase-3 and -9 cleavage. In addition, KPF has blocked the signaling of apoptosis mediated by c-Jun N-terminal kinase (JNK) and p38MAPK pathways, neutralizing ROS production (Filomeni et al., 2012). Mitochondrial autophagy and fission were also observed after KPF exposure, explaining the antiapoptotic effect and protective response (Filomeni et al., 2012).

#### Ischemia Stroke

Stroke is a neurological disorder, with an incidence which has grown every year associated with the aging of the population; more than 60% of the cases occur in individuals over 69 years old. It is classified as ischemic and hemorrhagic stroke (Manning et al., 2014; Thomalla et al., 2018).

Ischemic stroke is the most frequent form and, according to the World Stroke Organization (WSO), there are about 9.5 million new cases per year worldwide, of which 2.7 million (28.4%) die annually (WSO, 2016). Ischemic stroke is characterized by a blockage of the cerebral artery, usually caused by a clot (thrombus) which causes lack of oxygen and glucose in the brain, leading to brain cell death (Dawson and Dawson, 2017). Hemorrhagic stroke prevalence is in around 4.1 million people annually worldwide. However, the mortality rate is higher, approximately 2.1 million (51%) of the patients (WSO, 2016). Hemorrhagic vascular accident occurs through the rupture of a cerebral vessel, causing hemorrhage mostly occurring inside the brain tissue known as intraparenchymal tissue and usually associated with chronic arterial hypertension. Hemorrhagic stroke can occur on the brain surface, known as subarachnoid stroke and usually associated with an aneurysm rupture or uncontrolled hypertension (Smith and Eskey, 2011).

Currently, the therapeutic approach adopted for ischemic stroke is through the use of thrombolytic drugs, which must be administered within a time limit of 4.5 h after the onset of symptoms, although the patient may suffer damage to neurons and cells of the glia in the reperfusion process, caused mainly by the triggered inflammatory process and microglia activation (Dawson and Dawson, 2017). In contrast, hemorrhagic stroke requires a surgical approach in order to remove blood from the affected region and in some cases the placement of a catheter to relieve brain pressure (Smith and Eskey, 2011).

One recent study using an ischemic stroke model in rats by transient middle cerebral artery occlusion (MCAO) showed that the use of 100 mg/kg of KPF intragastrically administered for 7 days was able to inhibit microglia activation and expression of TNFα and interleukins such as IL-6, IL-1β, and IL-5. In addition, KPF inhibited MCP1 and ICAM1 proteins, both responsible for favoring the infiltration of immune system cells through the BBB. In contrast, KPF decreased MMP-3 expression, indicating BBB integrity promoted by KPF. Finally, KPF was responsible for significantly reducing the phosphorylation of NF kB p65 in addition to inhibiting its translocation to the nucleus (Li et al., 2019).

Wu et al. (2017) induced ischemic stroke by MCAO model in C57BL/6 mice treated with oral KPF for 7 days. The authors observed that KPF had a neuroprotective effect through inhibition of dynamin-related protein 1 (DRP1) phosphorylation, leading to a decrease in mitochondrial fission and affected mitochondrial morphology and functional integrity. In addition, they observed *in vitro* that primary neurons collected from the fetal cortex of rats exposed to succinate presented mitochondrial dysfunction. However, when the cells were treated with KPF for 7 days with concentrations between 100 and 200µM, it boosted the autophagy mechanism thereby protecting neurons against damage caused by glucose and oxygen deprivation.

KPF-3-O-rhamnoside (C_27_H_30_O_15_) and KPF-3-O-glucoside (C_21_H_20_O_11_) were evaluated in an *in vivo* MCAO-induced ischemic stroke model and both were capable of alleviating neurological deficits caused by ischemia, preventing neuron and axon damage. Effects were observed for intravenously administered doses of 10 mg/kg for KPF-3-O-rhamnoside and 7.5 mg/kg for KPF-3-O-glucoside. In addition, KPF-3-O-rhamnoside and KPF-3-O-glucoside inhibited microglia and astrocyte activation through inhibition of STAT3 phosphorylation and NF-kB p65 translocation to the nucleus, respectively. Both pathways are known to be responsible for proinflammatory gene activation. Furthermore, an improvement in BBB neurovascular dysfunction was observed (Yu et al., 2013).

The kaempferide-7-O-(4″-O-acetylrhamnosyl)-3-O-rutinoside (KPF-7-O-rutinoside) was evaluated using an *in vivo* MCAO ischemic model and the results showed that KPF-7-O-rutinoside was capable of reducing neurological deficit by oxidative stress inhibition, decreasing MDA levels and increasing GSH-PX and SOD activity (Wang et al., 2016). In addition, KPF-7-O-rutinoside inhibited NF-kB p65 phosphorylation, ICAM-1, COX-2, iNOS, and BAX2 levels, and caspase-3 and -9 cleavage, explaining the KPF-7-O-rutinoside neuroprotective, anti-inflammatory and antiapoptotic action.

To the best of our knowledge, there are no published studies reporting on KPF pharmacological action in hemorrhagic stroke.

#### Epilepsy

Epilepsy is a chronic neurological disorder, characterized by repeated seizures over an interval of more than 24 h, affecting people at any age, for which the causes are still unclear (Fisher et al., 2014). Currently, epilepsy affects more than 70 million people worldwide, with a high incidence mainly in children under 1 year of age and in adults over 50. For reasons not yet clarified, epilepsy is more frequent in low-income countries, with a rate above 80 cases per 100,000 people per year, unlike developed countries which have an incidence rate of around 50 cases per 100,000 people per year (Ngugi et al., 2011; Neligan et al., 2012; Bell et al., 2014; Fiest et al., 2017; Zack and Kobau, 2017; Thijs et al., 2019).

Little is known about the pathophysiology of epilepsy; however, it is believed that the onset of crises is due to the imbalance of the inhibitory action on neurotransmission of gamma-aminobutyric acid (GABA) and the excitatory one mediated by glutamate. In addition, epileptic seizures can be divided into focal seizure, when it affects only a set of neurons in part of the brain, or generalized seizure when it involves neuron dysfunction in both brain hemispheres (de Almeida et al., 2008).

Currently, there are several medications for the control of epileptic seizures. However, these medications cause great discomfort to patients due to their various side effects such as depression, impaired cognition, and decreased motor capacity (Ramalingam et al., 2013). In addition, about 30% of the affected patients have presented persistent seizures even with the use of antiepileptic medications (Ramalingam et al., 2013). Natural compounds have been an important source in the discovery of new drugs and can be a viable alternative for the discovery of new drugs with an antiepileptic action with milder side effects (Zhu et al., 2014). Studies have already shown that flavonoids have an antiepileptic capacity, as they have a structure similar to benzodiazepines, which suggests that these compounds can be better explored regarding their epilepsy treatment potential (Nassiri-Asl et al., 2008).

The glycoprotein P (gp-P) encoded by the *MDR1* gene is a transmembrane protein that has the function of protecting the cell against xenotoxins. In the brain, gp-P is expressed in astrocytes, neurons, and endothelial cells. The overexpression of this protein in the epileptogenic tissue is associated with resistance to antiepileptic drugs as it restricts the penetration of these drugs in the brain tissue (Dallas et al., 2006; Potschka and Brodie, 2012). Ferreira et al. (2018) proposed to evaluate the action of several flavonoids, including KPF, regarding their ability to reverse multidrug resistance mediated by gp-P. The authors showed that among 11 studied flavonoids, five KPF, at 200 μM concentration, were able to inhibit gp-P from Madin–Darby canine kidney (MDCK) cells, promoting the intracellular accumulation of antiepileptic drugs such as carbamazepine, phenytoin, oxcarbazepine, lamotrigine, and its active metabolites carbamazepine-10,11-epoxide and licarbazepine. However, it is not clear how KPF promotes the gp-P inhibition mechanism and further studies need to be done to clarify the KPF effect in epilepsy.

González-Trujano and collaborators (2017) have demonstrated in an *in vitro* study, using male Swiss Webster mice and Wistar rats induced to seizures through the pentylenetetrazol (PTZ), that the *Justicia spicigera Schltdl* extract containing Kaempferitrin as active component was able to significantly delay the onset of myoclonic seizures and generalized seizures at doses of 100 and 1,000 mg per kg, as well as tonic seizures at doses from 30 to 1,000 mg per kg. In addition, the study showed that the administration of Kaempferitrin intracerebroventricularly (1 mg/ml in the fourth ventricle), plus *Justicia spicigera Schltdl* administered intraperitoneally, improved the anticonvulsant activity already evident from the extract of *Justicia spicigera Schltdl*. In general, the study suggests that *Justicia spicigera Schltdl* has an anticonvulsant potential, being the Kaempferitrin as the compound partially responsible for the evidenced effects.

In addition, studies have showed a relationship between neuroinflammation and neurodegenerative diseases such as AD, PD, and epileptogenesis or the development of recurrent epileptic seizures (Vezzani et al., 2011; Zhang et al., 2013; Trovato Salinaro et al., 2018). Thus, an *in vitro* study with a tiliroside, a KPF derivative 3-O-[(E)-(2-oxo-4- (*p*-tolyl) but-3-en-1-yl] (2.5, 5, and 10 µM), using cell culture BV-2 mouse microglia evidenced an important inhibitory action in the production of proinflammatory mediators such as TNFα, IL-6, PGE2, and nitrite and reduced the levels of proinflammatory proteins COX-2 and iNOS. Also, tiliroside prevented the phosphorylation of p65 subunit of NF-kB, which is one of the critical steps in the microglia NF-kB activation pathway, after stimulation of BV-2 microglia with LPS and IFNγ. The mouse hippocampal HT22 neuron cell line in medium containing LPS + BV-2 stimulated by IFNγ resulted in reduced cell viability and generation of reactive species of oxygen, when these cells were exposed to tiliroside, indicating no damage to neural cells or generation of oxidative stress, besides having promoted the activity of the AMPK and Nrf2/HO-1 pathways responsible for suppressing neuroinflammation (Velagapudi et al., 2019).

Fewer studies have evaluated the isolated KPF effect in epilepsy; however, KPF does seem to be a potential pharmacological treatment for epilepsy.

#### Major Depressive Disorder

MDD is a debilitating mental disorder difficult to treat and negatively impacts affected individuals (Kessler et al., 2005). The WHO defines MDD as a mental disorder characterized by persistent sadness and the loss of interest in activities that are normally pleasurable, accompanied by the inability to perform daily activities for at least two weeks (Reddy, 2010). Commonly affected individuals have symptoms such as loss of concentration, changes in sleep and appetite, guilt, suicidal thoughts, and/or the intention of causing harm to themselves (Kessler et al., 2005). Approximately 5% of the world population, about 350 million people, present MDD and, for each man, two women are affected (WHO, 2014; WHO, 2020). Moreover, data from the National Violent Death Reporting System (NVDRS), including 32 American states, have shown that the majority of deaths in the year 2016 were suicide (62.3%) due to the MDD (Ertl et al., 2019).

Initially, the deficiency of monoamine neurotransmitters in depressed patients, mainly serotonin, norepinephrine, and dopamine, has given rise to the first monoaminergic drugs (Gerhard et al., 2016). Then the DDM treatment evolved to the tetracyclic antidepressants (TCAs), which mostly act by blocking norepinephrine, serotonin, and dopamine receptors and the monoaminooxidase inhibitor antidepressants (MAOIs), which reduce the activity of the monoaminooxidase enzyme (MAO) resulting in an increase in serotonergic, noradrenergic, and dopaminergic neurotransmitters in the CNS (Harrigan and Brady, 1999; Gerhard et al., 2016). However, MAOIs may cause fatal complications when associated with foods rich in tyramine as cheeses and fermented foods, resulting in hypertension, heart attack, and stroke (Gerhard et al., 2016).

In order to have fewer side effects and, consequently, lower rate of treatment discontinuation, drug development focused on increasingly selective reuptake inhibitors (Birmes et al., 2003; Gerhard et al., 2016) as serotonin and norepinephrine reuptake inhibitors (SNRIs) and norepinephrine and dopamine reuptake inhibitors (NDRIs), by blocking the action of the norepinephrine transporter. This in turn leads to increased extracellular concentrations of norepinephrine and epinephrine and therefore can increase adrenergic neurotransmission (Brimes et al., 2003).

Some studies have indicated that oxidative stress and neuroinflammation may play a significant role in the pathogenesis of MDD. It was observed that MDD patients have an increase in oxidative damage, as well as decreased levels of endogenous antioxidants, in addition to an increase in inflammatory mediators such as IL-1β and TNFα (Gerhard et al., 2016). In animal models studies, behaviors like depression correlated with increased levels of inflammatory factors (Gerhard et al., 2016).

Despite the existence of treatments for the disorder, approximately one-third of individuals diagnosed with MDD do not respond to two or more antidepressant drugs and are characterized by treatment-resistant depression (Trivedi et al., 2006). More recently, Gao and collaborators (2019) have evidenced that KPF exerted antidepressant effects mediated by its antioxidant and anti-inflammatory capacity by increasing the AKT/β-catenin cascade in the prefrontal cortex. The study was evaluated in male C57 and CD1 mice at 8 weeks of age, using the model of chronic social defeat stress (CSDS), the action of KPF in two groups (10 and 20 mg/kg KPF). It was possible to observe a behavioral improvement in mice exposed to KPF. In addition, when the expression of phosphorylated AKT and β-catenin proteins in the prefrontal cortex after CSDS was evaluated, these were at increased levels in mice exposed to KPF. Furthermore, KPF reduced the concentrations of inflammatory mediators IL-1β and TNFα in the prefrontal cortex. The observed effects were more accentuated in mice exposed to a dose of 20 mg/kg of KPF; however, the effects were observed in both groups.

Garza et al. (2019) have evaluated the antidepressant effects of the KPF-3-O-glucoside and narirutin flavonoids through maternal supplementation in female wild Wistar rats. The rats were divided into five groups with different obesogenic diets. Only one group has also received supplementation with KPF-3-O-glucoside (15 mg/kg body weight [bw]) and narirutin (30 mg/kg bw) 3 weeks before mating. The pregnant rats remained on their respective diets until the birth of the offspring and lactation. It is important to note that maternal overnutrition during the perinatal period increases the susceptibility to behavioral changes in humans and animal models. The study demonstrated that the different hypercaloric diets used in the stimulation promoted behavioral changes in the female offspring, like depressive behaviors. However, the group supplemented with flavonoids have shown an important antidepressant modulation. It was also observed that the group of female offspring who received flavonoids supplementation reversed the insensitivity to the antidepressant imipramine, observed in the other groups.

#### Anxiety Disorders

The Diagnostic and Statistical Manual of Mental Disorders (DSM-5) recognizes AD as disorders that share characteristics of excessive fear and anxiety and related behavioral disorders and it can also be defined as the anticipation of a future threat (American Psychiatric Association, 2013). In addition, the DSM-5 recognizes AD as the anxiety disorder separation, the selective mutism, specific phobias, the social anxiety disorder, the panic disorder, agoraphobia, the generalized anxiety disorder, substance- and/or medication-induced anxiety, and anxiety due to other drug conditions (American Psychiatric Association, 2013). Unlike anxiety and adaptive fear, in AD these characteristics are excessive or persistent for a period beyond the appropriate period (American Psychiatric Association, 2013).

The WHO points to an AD worldwide prevalence around 3.6% and in Brazil the prevalence rises to 9.3%, being the country with the highest AD cases in the world (World Health Organization, 2017). Several conditions can lead to the emergence of anxiety; however, risk factors arising from work activity and relationships built on work have been one of the main causes, such as competitiveness in the labor market coupled with fear of unemployment leading people to precarious working conditions, which favors the emergence of mental disorders such as AD and MDD (Grupe and Nitschke, 2013).

The main kind of treatment besides psychotherapy is through TCAs, MAOIs, SNRIs, NDRIs, and Benzodiazepines drugs, which act by increasing the affinity of the GABA by it receptor, resulting in increased CNS inhibitory synaptic response (Murrough et al., 2015).

Gupta and collaborators (2018) have isolated bioactive compounds from the *Citrus paradisi* leaves, including KPF, and evaluated the KPF anxiolytic action or only the plant extract using both sexes of Swiss albino mice, which were submitted to different models of mental behavior of anxiety known as open field elevated plus maze (EPM), elevated plus maze model, and hole edge. Interestingly, an anxiolytic activity with 100 mg/kg bw of KPF or extract was observed, through the observation of an increase in permanence in the open arms in the EPM test, similar to mice exposed to diazepam.

Ahmad and collaborators (2020) have used *in vivo* and *in vitro* models to demonstrate the anxiolytic action performed by KPF. Thus, the *in vitro* test, using a fatty acid amide hydrolase (FAAH) inhibitor screening assay and 0.1–200 µM KPF concentration, showed that KPF was able to inhibit the enzyme FAAH responsible for regulating the duration of the action of the endocannabinoid molecule (eCB) system, which regulates complex circuits involved in the central role play in states similar to anxiety. In addition, eCB system has been associated with several psychic disorders. The KPF regulation occurred in a concentration-dependent manner (IC50: 1.064 µM) and promoted an increase in eCB activity. *In vivo* results using adult Wistar rats subjected to an EPM protocol plus light impulses by short electric shock in the rats’ foot demonstrated that animals treated with KPF (40 mg/kg) presented reduced fear reaction as freezing response.

#### Neuropathic Pain

Neuropathic pain is defined by the International Association for the Study of Pain (IASP) as pain generated through an abrasion or dysfunction in the nervous system (Merskey and Bogduk, 1994). Several factors can damage the nerves in the peripheral nervous system (PNS) or CNS culminating in neuropathic pain (Baron et al., 2010). In addition, several diseases can lead to this condition, such as autoimmune diseases (for example, multiple sclerosis), metabolic diseases (for example, diabetic neuropathy), infections (herpes zoster), vascular diseases (strokes), and cancer. Neuropathic pain affects about 10% of the world population and can be divided into mononeuropathic, in which one nervous path is compromised, polyneuropathic, when pain is well located, and generalized, when several nerves are damaged (Campbell and Meyer, 2006; Baron et al., 2010).

The treatment for neuropathic pain is mostly focused on treating the symptoms; except under specific conditions, the etiological causes can be treated and thus relieve the pain (Campbell and Meyer, 2006; Baron et al., 2010). However, currently treatments available have a moderate effectiveness and increased side effects (Baron et al., 2010).

In our best knowledge, there are no studies showing the isolated action of KPF in neuropathic pain. On the other hand, two studies have evaluated total plant extracts, which contain KPF in their composition (Raafat and El-Lakany, 2015; Singh et al., 2017). Thus, Raafat and El-Lakany (2015) have shown that *Ferula hermonis L.* at dosages of 12, 5, 25, and 50 mg per kg and *Sambucus nigra L.* using 50, 100, and 200 mg per kg of extracts exercised hypoglycemic activity in diabetes mellitus animal models and improved the peripheral nervous function of animals, suggesting that the antioxidant action of KPF prevented oxidative stress and it may have contributed significantly to the antinociceptive effect. Singh et al. (2017) have shown that the *Alstonia scholaris* extracts (100 and 200 mg per kg) were able to improve the neuropathic pain induced in mice through a chronic sciatic nerve constriction injury. The study highlighted several types of flavonoids present in *Alstonia scholaris*, emphasizing the higher KPF concentration. The authors also attributed the improved pain effect to the antioxidant and anti-inflammatory action of KPF.

#### Glioblastoma

CNS tumors are a disease group that presents a disordered growth of cells that make up the brain and spinal cord (Howlader et al., 2011). CNS tumors are associated to high morbidity and mortality rates in affected patients, being the main cause of cancer death in early childhood. In children under 15, CNS tumors are the second most frequent, only surpassed by hematological neoplasms (Howlader et al., 2011). The World Health Organization (WHO) estimates there will be 309,040 new cases in the world in 2020, with a mortality rate of approximately 81% (251,964) (Bray et al., 2018). In Brazil, 12,955 new cases are expected in the same year and a mortality rate of around 87% (11,323) (Bray et al., 2018).

In fact, CNS tumors are multifactorial being the result of several inherited or acquired genetic alterations due to exposure to environmental factors such as ionizing radiation, carcinogenic agents, tobacco, and alkylating agents, as well as immune disorders caused by the human immunodeficiency virus or immunosuppressive drugs (Bondy et al., 2008).

Gliomas are the most frequent CNS tumors, totalizing 50% of the cases. They result from glial cells classified according to their cellular and molecular origins (Louis et al., 2016). Glial cells, subdivided into astrocytes, oligodendrocytes, and ependymal cells, constitute approximately 90% of the cells that make up the brain, and mutations in glial cells or in their precursor cells can trigger the appearance of gliomas, subdivided into astrocytomas, oligodendrogliomas, and ependymomas (Gomes et al., 2013).

Astrocytomas represent about 30% of gliomas and they can be classified into degrees of malignancy, according to WHO as pilocytic astrocytoma (grade I), diffuse astrocytoma (grade II), anaplastic astrocytoma (grade III), and GBM (grade IV). GBM is the most frequent and aggressive, representing 80% of the malignant gliomas cases (Badke et al., 2014; Louis et al., 2016).

Santos and collaborators (2015) have evaluated KPF effect on the GMB cell line, GL-15, and the authors observed that KPF at 50 µM for 48 h was able to inhibit the activity of the metalloproteinases MMP2 and MMP9, which is associated with the aggressiveness of these tumors. There was also a noticeable increase in laminin and fibronectin proteins expression in the GBM extracellular matrix. It is known that higher MMP2 and MMP9 and lower laminin and fibronectin are associated with migration and invasion of tumor cells (Santos et al., 2015). In addition, KPF promoted apoptotic morphological cell changes, although no significant cell viability inhibition was observed by 3-[4,5-dimethylthiazol-2-yl]-2,5-diphenyltetrazolium (MTT) assay.

One study has evaluated KPF action *in vitro* using the GBM cell line GBM8401 exposed to 12-otetradecanoylpholia-13-acetate (TPA) at 5, 10, 20, and 40 μM, a MMP9 inducer activity, and consequent cell migration and invasion through kinase C α protein (PKCα), extracellular signal-regulated kinases (ERK), and NF-kB pathways activation. When cells were exposed to KPF, there was significant decrease in PKCα protein and ERK and NF-kB pathways inhibition occurred with consequently lower MMP9 expression followed by a reduction of invasion and migration cells (Lin et al., 2010).

### Kaempferol: *In Vivo* Rodent Magnetic Resonance Imaging Studies

Local control of calcium (Ca2+) in subcellular compartments influences CNS metabolism and communication. Ca2+ waves propagate along individual cells and through network of connected cells of neuronal and glial types integrated by various cytoplasmic Ca2+ buffering proteins and subcellular organelles such as the mitochondria. Mitochondria take up Ca2+ primarily through a uniporter mechanism buffering some of it and efflux of the excess free Ca2+. Novel biophysical approaches in the past few decades have revealed the integrating role of mitochondrial Ca2+ cycling in neural metabolism (Duchen, 1992) and signaling (Mann et al., 2009). Using *in vivo* rat model, it has been recently shown that Ca2+ uniporter activity was enhanced using KPF at 25 mg per kg and 30 μM in the animals blood plasma, by mitochondrial and overall cytoplasmic Ca2+ levels increasing, indicating that mitochondrial Ca2+ uptake was modulated by KPF treatment (Sanganahalli et al., 2013; Kannurpatti et al., 2015). In addition, same group has shown neuroprotective effects of KPF in traumatic brain injury (TBI) rats. These studies showed that TBI prognosis was significantly altered at adolescence by early KPF treatment, with improved neural connectivity, neurovascular coupling, and parenchymal microstructure in selected brain regions. However, KPF failed to improve vasomotive function across the whole brain, as measured by cerebrovascular reactivity. The differential effects of KPF treatment on various brain functional compartments support diverse cellular-level mitochondrial functional outcomes *in vivo* (Parent et al., 2019).

## Conclusion

KPF has shown an important neuroprotective action in all addressed diseases, mainly promoting an anti-inflammatory and antioxidant effect. In addition, KPF promoted a protective effect on the brain, inhibiting proinflammatory cytotoxicity and the activity of important inflammatory pathways as NF-kB, p38MAPK, and AKT, resulting in an overall anti-inflammatory action. In conclusion, we suggest that KPF and some glycosylated derivatives (KPF-3-O-rhamnoside, KPF-3-O-glucoside, KPF-7-O-rutinoside, and KPF-4′-methyl ether) have multipotential neuroprotective actions in the CNS diseases. Overall, the lack of *in vivo* data makes it difficult to construct broad generalizations concerning the benefits of KPF in neurological diseases. Thus, the present review may contribute to the field and future *in vivo* studies may clarify the mechanisms of KPF action in CNS diseases as well as the impact of glycosylation on KPF bioactivity.

## Author Contributions

JS, PO, and MO wrote and revised the manuscript; JG performed all molecule designs.

## Conflict of Interest

The authors declare that the research was conducted in the absence of any commercial or financial relationships that could be construed as a potential conflict of interest.
